# Supplementary material for: Enabling liquid crystal elastomers with tunable actuation temperature
Source: Nat Commun. 2023 Jun 14;14:3518. doi: 10.1038/s41467-023-39238-2 (PMC10267162; doi:10.1038/s41467-023-39238-2)
Supplement: Supplementary file 1 — Supplementary Information [file 41467_2023_39238_MOESM1_ESM.pdf]

Supplementary Information For Enabling liquid crystal elastomers with tunable actuation temperature

Yanjin Yao[1], Enjian He[1], Hongtu Xu[1], Yawen Liu[1], Zhijun Yang[1], Yen Wei[1,2] & Yan Ji[1]*

[1]The Key Laboratory of Bioorganic Phosphorus Chemistry & Chemical Biology (Ministry of Education), Department of Chemistry, Tsinghua University, Beijing 100084, China

[2]Department of Chemistry, Center for Nanotechnology and Institute of Biomedical Technology, Chung-Yuan Christian University, Chung-Li 32023, Taiwan, China

E-mail: jiyan@mail.tsinghua.edu.cn

Materials

Diglycidyl ether of 4, 4′- dihydroxybiphenyl (DGE-BP) and 4′-dihydroxy-α-methylstibene (DGE-DHMS) were synthesized following in a typical approach.[1] RM82 (97%) and 1,4-Bis-[4-(3-acryloyloxypropyloxy) benzoyloxy]-2-methylbenzene (RM 257, 98%) were purchased from Shijiazhuang Sdynano Fine Chemicals. Lysine triisocyanate (85%) was purchased from Zhengzhou JACS Chem Product Company. Hydrochloric acid (HCl, AR) and tetrahydrofuran (THF AR) and anhydrous dichloromethane (DCM AR) were purchased from Beijing Tongguang Fine Chemicals. Sodium chloride (NaCl; >99.9%) was purchased from Shanghai Tian Scientific Company. Sylgard184, a silicone elastomer kit, was purchased from Dow Corning (Midland, MI). Sebacic acid (TCI, 99.0%), triazabicyclodecene (TCI, 98%), Poly(ethylene glycol)diol (Mn:400, TCI,98%), 2-Mercaptoethanol (TCL 98%), trimethylamine (TCL 98%), Irgacure-651 (I 651, 98%), n-butylamine (n-BA, 99.5%, J&K), Hexamethylene diisocyanate (98% J&K), Dibutyltin dilaurate (DBTDL, 95% Adamasbeta), Glycerin (Aladdin, ≥99.5%), diglycidyl ether of bisphenol A (DGE-BA) (Sigma-aldrich, D.E.R. 332), and 1, 3-Dimercaptopropane (98% Energy Chemical) were used directly without further purification.

Synthesis

Synthesis of xLCE-BP

Similar to our previous work[1] we prepared xLCE-BP by reacting diglycidyl ether of 4, 4'-dihydroxybiphenol（DGE-DHBP, 2.384 g, 8.00 mmol) with sebacic acid (1.616 g, 8.00mmol) at 170°C. Triazabicyclodecene catalyst (0.25 mol% to the COOH groups) was introduced and stirred manually until homogeneous. It was cooled to room temperature when the mixture became very viscous. Then the mixture was sandwiched between two plates to be cured by a hot press with 3 MPa pressure for certain time at 170°C. To make sure of complete crosslinking, the curing time in this work was 6 hours (h) unless otherwise noted.

Synthesis of xLCE-DHMS

As shown in our previous work[2], we prepared xLCE-DHMS by reacting 4, 4′-dihydroxy-α-methylstibene (DGE-DHMS：2.72 g, 8.00 mmol) with sebacic acid (1.616 g, 8.00mmol) at 160°C. A triazabicyclodecene catalyst (0.25 mol% to the COOH groups) was introduced and stirred manually until homogeneous. It was cooled to room temperature when the mixture became very viscous but was not completely cross-linked. Then the mixture was sandwiched between two plates to be cured by a hot press with 3 MPa pressure for 6 h hours at 160°C.

Synthesis of xLCE-PU

The fully cured polydomain xLCE-PU samples were prepared by a classic "one-pot" reaction between hydroxyl and isocyanate.[3] Hydroxyl terminated LC-oligomer (0.47 g, 0.1 mmol, 1 eq.) and lysine triisocyanate (cross-linker) (0.031 g, 0.1 mmol, 0.1 eq.) were dissolved in 2 ml of DCM (the solvent should be used as little as possible to ensure that the system has relatively high viscosity). After that, 0.25 wt % DBTDL was added to initiate the crosslinking process. The mixture was stirred at room temperature, poured into a custom-made Teflon mold (4.0 cm long × 4.0 cm wide × 1.0 cm deep), reacted for 12 h at 25°C, and further cross-linked for 3 h at 100°C. After that, all the samples were immersed in DCM to extract soluble contents. The final polydomain xLCE-PU samples were obtained after drying in a vacuum oven at 30°C for 24 h.

Synthesis of xLCE-RM257

xLCE-RM257 was synthesized with reference to our previous work.[4] The specific synthesis process is as follows. Synthesis of the diacrylate end-capped liquid-crystalline oligomers (LCOs): RM257 and chain-extender (n-BA) with required ratio of diacrylate: amine (1.15: 1) were individually added into 25 mL round-bottomed flasks. The mixtures were heated with stirring at 85°C for 20 h, during which, the acrylate and amine groups underwent chain extension via aza-Michael step-growth addition reactions, resulting inLCO-15%. Synthesis of xLCE-RM257: The aforementioned as-prepared three types of LCOs (0.4 g) and the photoinitiator I 651 (3 wt% of the total LCOs) were individually added into 2 mL centrifuge tubes. Also, 1 mL tetrahydrofuran was added to dissolve the mixtures with ultrasound dispersion. Then the viscous mixtures were transferred into home-made quartz glass molds (assembling two pieces of quartz glass separated by a 400 μm spacer), followed by being exposed to UV light (λ = 365 nm, 40 mW cm^2 and 20 min for each side) at room temperature for photopolymerization of the acrylate groups. After photopolymerization, the obtained xLCE-RM257 were dried in a vacuum oven at 60°C for 24 h.

Synthesis of Vitrimer-BA

As shown in our previous work[5], we prepared Vitrimer-BA by reacting diglycidyl ether of bisphenol A（DGE-BA：2.72 g, 8.00 mmol) with sebacic acid (1.616 g, 8.00mmol) at 160°C, and a triazabicyclodecene catalyst (0.25 mol% to the COOH groups) was introduced and stirred manually until homogeneous. It was cooled to room temperature when the mixture became very viscous but was not completely cross-linked. Then the mixture was sandwiched between two plates to be cured by a hot press with 3 MPa pressure for 6 h hours at 160°C.

Synthesis of Vitrimer-PU

The Vitrimer-PU was synthesized by reacting poly(ethylene glycol)diol (Mn: 400) and glycerin with hexamethylene diisocyanate in the presence of dibutyltin dilaurate(DBTDL) as a catalyst for transcarbamoylation according to previous reference.[6] The reaction was carried out in tetrahydrofuran solvent.

Synthesis of PDMS

The PDMS elastomer disk was prepared from Dow Corning's Sylgard 184 elastomer kit as described in the literature.[7] The two-part liquid components of the kit were mixed in a 10:1 ratio by weight following the manufacturer's instructions. The mixture was mixed manually for 5 min. To cast the elastomer disk, the mixed liquid (5± 0.05 g) was poured into a custom-made Teflon mold (4.0cm long × 4.0cm wide × 1.0cm deep) and then the Teflon mold was placed on a level surface and cured at 100°C in a vacuum oven for 24 h. The cured PDMS elastomer film (～0.26 mm thick) was peeled from the Teflon mold.

Investigation on the curing time of xLCE-BP.

To find out the suitable curing time, the starting materials for the synthesis of xLCE-BP were mixed and cured for 3 h, 9 h, 12 h, and 15 h, respectively.

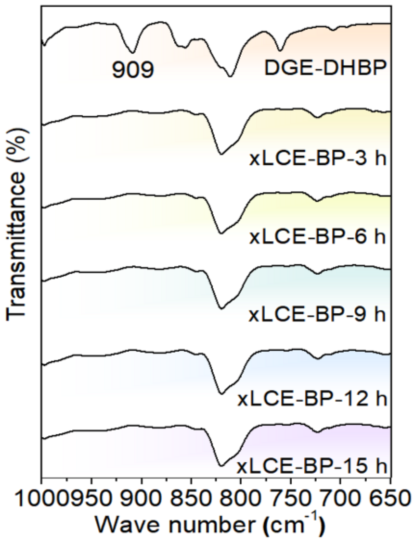

Figure S1. FTIR spectra for xLCE-BP with different curing time.

Swelling experiments were done on xLCE-BP with different curing times to further confirm the covalent crosslinking. Samples were immersed in 20 mL DCM at room temperature, the volume changes of the samples were recorded at 6 h, 24 h, and 5 days (d) intervals, and a new solvent was used to replace the old one at the same intervals. The results showed that the volume of all samples almost reached the maximum volume after swelling for 6 h. To ensure that the unreacted small molecules had been removed, all the samples in our paper were swollen for 24 h.

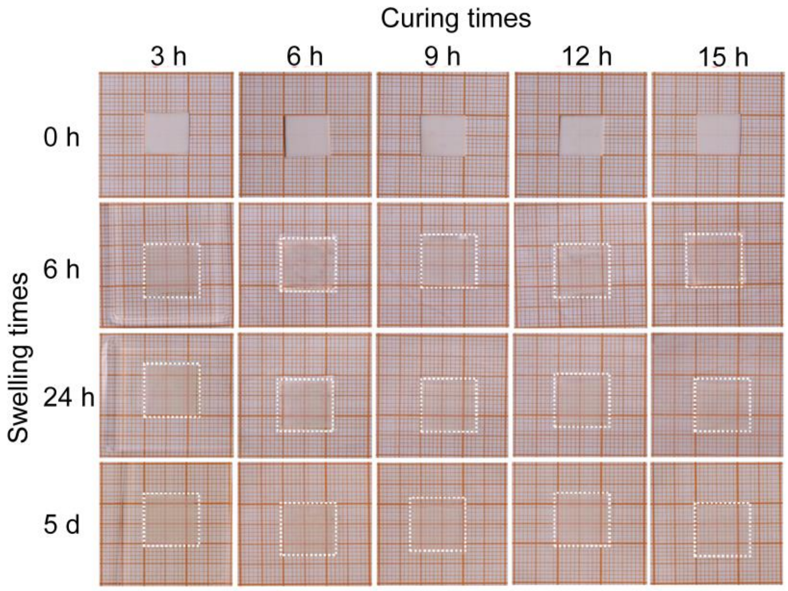

Figure S2. Swelling tests for xLCE-BP with different curing time.

TGA was used to further characterize the thermal stability of xLCE-BP samples with different curing time.

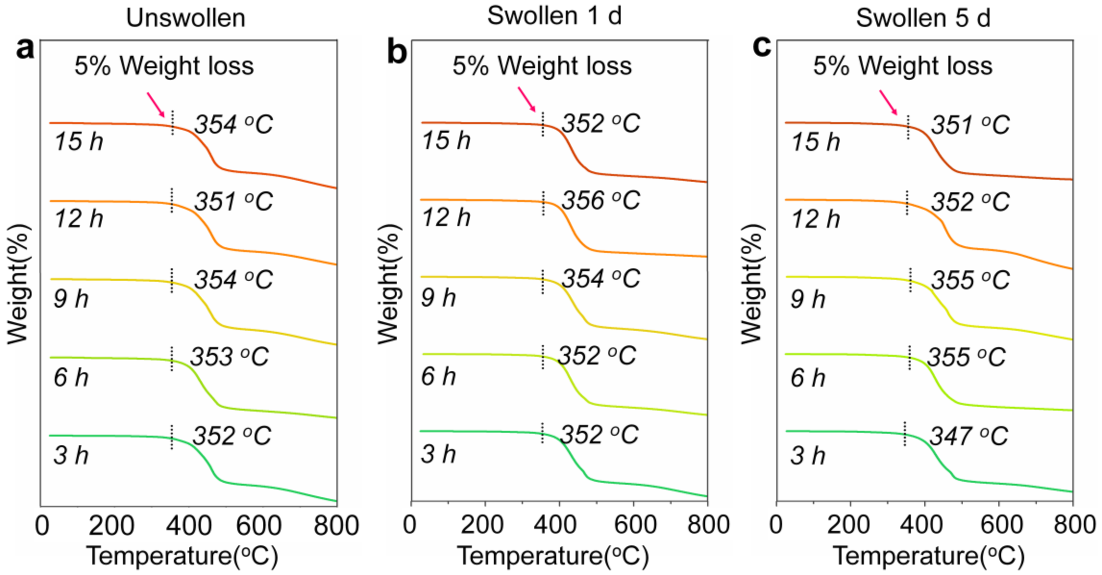

Figure S3. TGA for xLCE-BP (a) unswollen; (b) swollen for 1 d; (c) swollen for 5 d.

Preparation of monodomain liquid crystal elastomers with different actuation temperatures

All the monodomain LCEs were prepared from polydomain xLCE-BP (T_{i0}= 114°C) by annealing at different temperatures for different time.

(1)Preparation of the monodomain xLCE-BP with a T_i of 90°C: The polydomain xLCE-BP (T_{i0}= 114°C) sample was stretched to 220% of the original length, then fixed at 20% of the original length, and annealed in a 180°C vacuum oven for 24 h. It was then heated to 120°C to remove the elastic deformation. After cooling down, a monodomain sample with a T_i of 90°C was obtained.

(2) Preparation of the monodomain xLCE-BP with a T_i of 112°C: The polydomain xLCE-BP (T_{i0}= 114°C) sample was stretched to 220% of the original length, then fixed at 20% of the original length, and annealed in a 180°C vacuum oven for 3 h. It was then heated to 120°C to remove the elastic deformation. After cooling down, a monodomain sample with a T_i of 112°C was obtained.

(2) Preparation of the monodomain xLCE-BP with a T_i of 122°C: The polydomain xLCE-BP (T_{i0}= 114°C) sample was stretched to 220% of the original length, then fixed at 220% of the original length, and annealed in a 110°C vacuum oven for 3 d. It was then heated to 130°C to remove the elastic deformation. After cooling down, a monodomain sample with a T_i of 122°C was obtained.

(3) Preparation of the monodomain xLCE-BP with a T_i of 135°C: The polydomain xLCE-BP (T_{i0}= 114°C) sample was stretched to 220% of the original length, then fixed at 220% of the original length, and annealed in a 110°C vacuum oven for 10 d. It was then heated to 140°C to remove the elastic deformation. After cooling down, a monodomain sample with a T_i of 135°C was obtained.

(4) Preparation of the monodomain with a T_i at 150°C: The polydomain xLCE-BP (T_{i0}= 114°C) sample was stretched to 220% of the original length, then fixed at 220% of the original length, and annealed in a 110°C vacuum oven for 30 d. It was then heated to 160°C to remove the elastic deformation. After cooling down, a monodomain sample with a T_i of 150°C was obtained.

Patterning of unswollen xLCE-BP films (T_{i0}= 113°C)

To get the patterned films in Figure 5a, we designed five patterns with CAD software. Polyimide electrothermal films were prepared according to those patterns and directly pasted on the xLCE-BP polymer film with a length * width * thickness of 10cm*10cm*0.26mm. The temperature is set to 105°C by adjusting the voltage of the external circuit. After heating at 105°C for 3 d, the heating temperature was adjusted to 110°C. The infrared images of the pattern during heating are shown in Figure 5a (middle row). After heating the sample for 10 days and removing the heating film, the T_i of the heated area is 135°C while the T_i of the unheated part is 113°C.

To get the patterned films in Figure 5b, the same method was used as the above except that the pattern and the heating time varied. All the patterns were heated at 105°C for 3d, “2” and”3” were further heated at 110°C for 10 d. “4”, “5” and “6” were further heated at 110°C for 40 d. The T_i of the unheated part of the film is 113°C, the T_i of pattern “1” is 125°C, the T_i of pattern “2” and “3” is 135°C, the T_i of pattern “4”, “5” and “6” is 152°C, respectively.

Characterization

Fourier transform infrared (FTIR) spectra were obtained on Perkin Elmer spectrum 100. Differential scanning calorimetry (DSC) experiments were performed using the TA-Q2000 DSC apparatus at a heating or cooling rate of 5ºC min^{-1} under nitrogen flow. The measurement procedure consists of two scan cycles, where both the heating and cooling rates were. The first scan was to eliminate the thermal history of the sample, and the data of the second scan was plotted to determine T_i and T_g. Thermogravimetric analysis (TGA) results were obtained on TA instruments Q50 under a nitrogen atmosphere with a heating rate of 10°C min^{-1} from room temperature to 800°C. Dynamic mechanical analysis was used to evaluate the actuation stability for the monodomain of xLCE-BP. It was performed on a TA instruments Q800 dynamic mechanical analyzer (DMA) apparatus in the tension film geometry under the controlled force mode. 2D X-ray diffraction (XRD) images were obtained on the Instrument of SAXSLAB Ganesha, and its diffractometer with a wavelength of 0.154 nm.

Table S1 The values of T_i for the swollen-polydomain xLCE-BP (T_{i0} = 114ºC) after annealing at 110ºC for different time.

Annealing Time	Swollen-polydomain (xLCE-BP)	
	T_i (ºC)	
	Heating (5ºC/min)	Coolimg (5ºC/min)
0d	114	108
3d	131	120
5d	145	128
10d	155	138
15d	165	150
20d	167	152
25d	170	158
30d	173	163
35d	174	158
40d	174	159
45d	176	163
50d	178	166

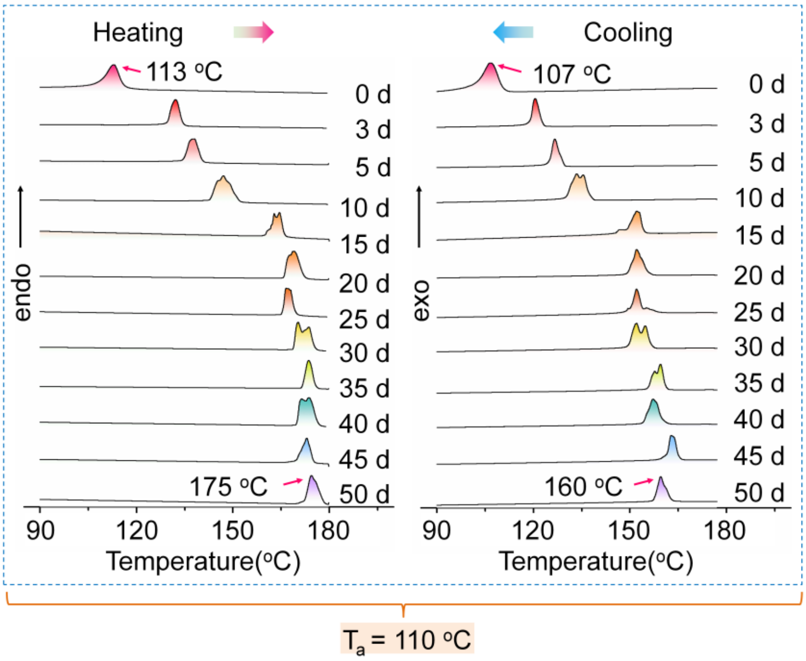

Figure S4. DSC traces of unswollen polydomain xLCE-BP annealed at 110°C at different time.

Table S2 The values of T_i for the unswollen-polydomain xLCE-BP (T_{i0} = 113°C) after annealing at 110°C for different time.

Annealing Time	T_i (°C)	
	unSwollen-polydomain (xLCE-BP)	
	Heating (5°C/min)	Coolimg (5°C/min)
0d	113	107
3d	131	121
5d	137	127
10d	147	135
15d	165	152
20d	169	152
25d	168	152
30d	174	155
35d	174	160
40d	174	158
45d	173	163
50d	174	160

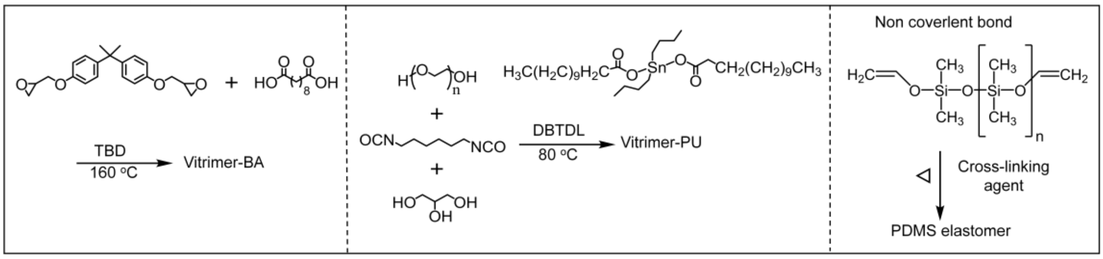

Figure S5. Synthesis of non-LC vitrimers with different dynamic covalent bonds (Vitrimer-BA and Vitrimer-PU) and PDMS elastomer without dynamic covalent bonds.

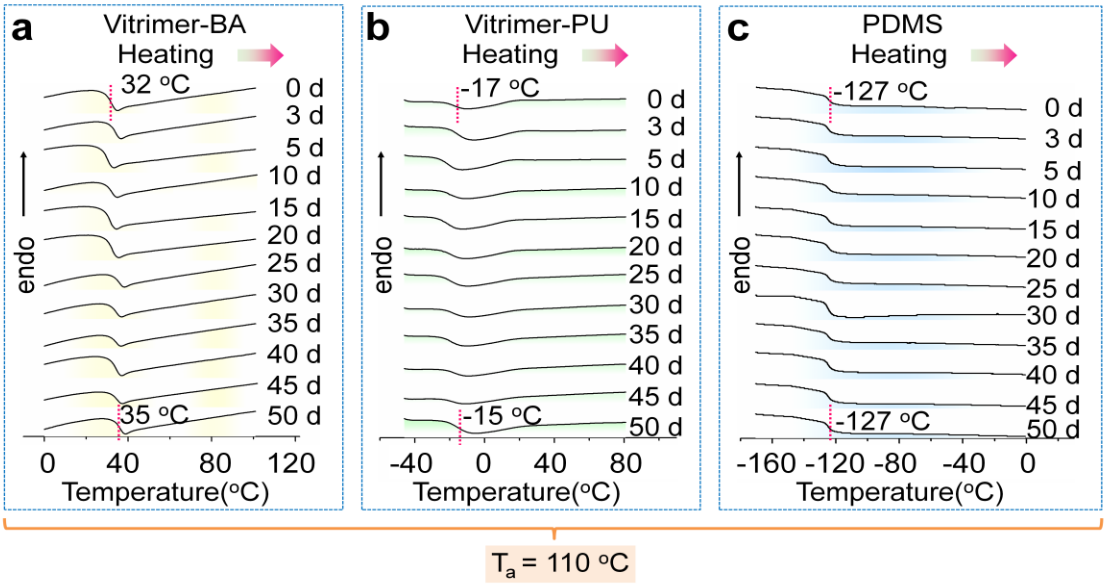

Figure S6. The DSC heating traces were used to determine T_g for Vitrimer-BA(a), Vitrimer-PU(b), and, PDMS elastomer after annealing at 110°C for different time.

Table S3 The values of T_g for polydomain xLCE-BP, PDMS elastomer, Vitrimer-PU, and Vitrimer-BA after annealing at 110°C for different time. T_g is determined from the heating trace.

Annealing Time	T_g (°C)			
	(xLCE-BP)	(Vitrimer-BA)	(Vitrimer-PU)	(PDMS)
0d	58	32	-17	-127
3d	65	33	-15	-127
5d	67	30	-18	-127
10d	69	32	-17	-127
15d	69	31	-19	-126
20d	69	32	-17	-127
25d	72	34	-17	-127
30d	71	33	-16	-127
35d	73	33	-18	-126
40d	72	33	-16	-126
45d	71	33	-17	-127
50d	74	35	-15	-127

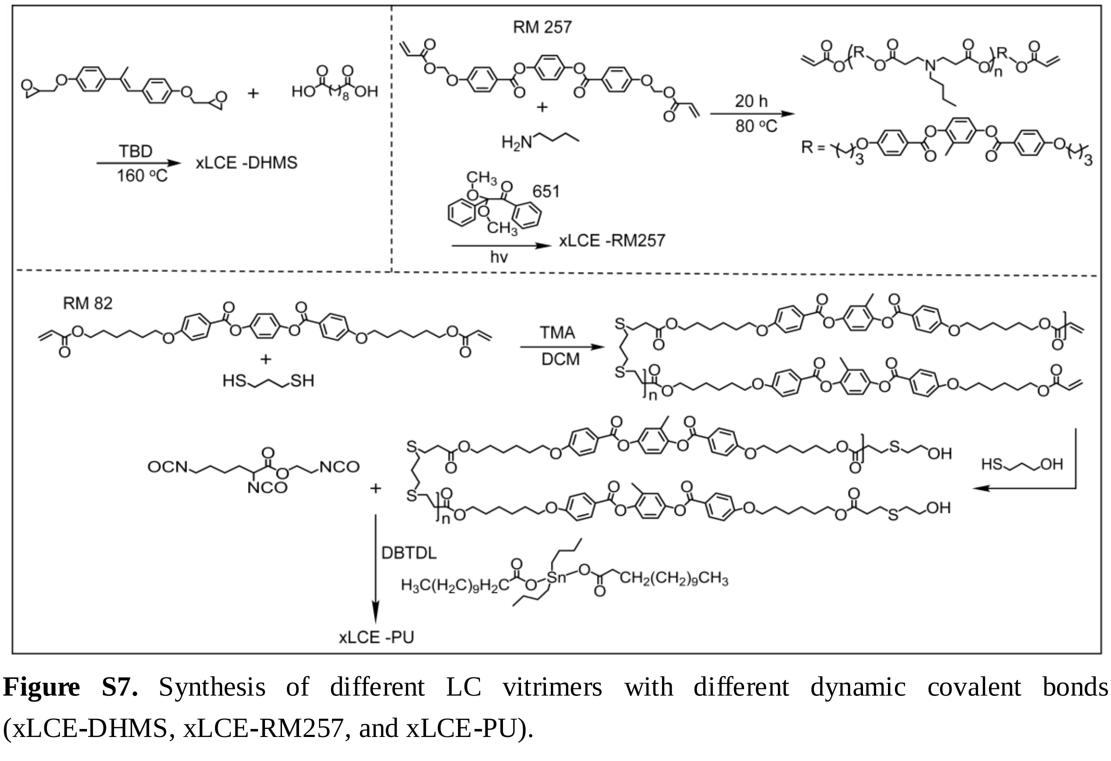

Figure S7. Synthesis of different LC vitrimers with different dynamic covalent bonds (xLCE-DHMS, xLCE-RM257, and xLCE-PU).

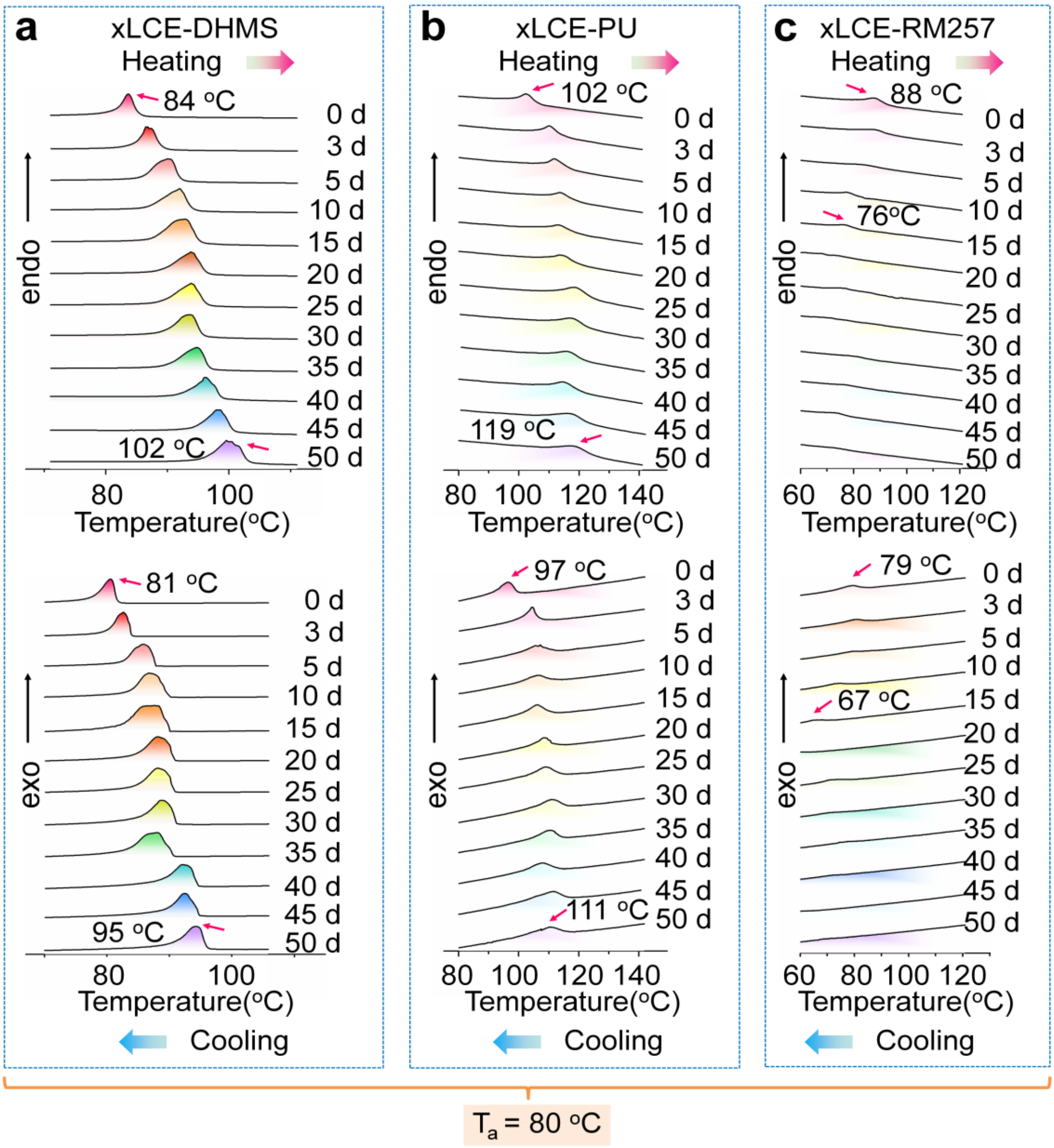

Figure S8. DSC traces of both heating/cooling (rate of 5ºC/min) of xLCE-DHMS (a), xLCE-PU (b), xLCE-RM257 (c).

Table S4 The values of T_i for xLCE-DHMS, xLCE-PU, and xLCE-RM257 after annealing at 80°C for different time.

Annealing Time	T_i (°C)					
	Swollen-polydomain (xLCE-DHMS)		Swollen-polydomain (xLCE-PU)		Swollen-polydomain (xLCE-RM257)	
	Heating (5°C/min)	Coolimg (5°C/min)	Heating (5°C/min)	Coolimg (5°C/min)	Heating (5°C/min)	Coolimg (5°C/min)
0d	84	81	102	96	88	79
3d	87	83	110	104	87	79
5d	91	86	112	106	83	78
10d	92	87	114	107	78	72
15d	93	88	114	106	76	67
20d	94	89	114	109	/	/
25d	94	89	117	109	/	/
30d	94	90	118	111	/	/
35d	95	88	116	111	/	/
40d	97	93	116	108	/	/
45d	99	93	118	112	/	/
50d	102	95	119	111	/	/

Table S5 The values of T_i for swollen-monodomain xLCE-BP after annealing at 110°C for different time. As the monodomain samples were obtained by annealing the stretched polydomain xLCE-BP, there is no monodomain xLCE-BP at 0d. Therefore, the T_i data of polydomain xLCE-BP before annealing were used for 0d.

Annealing Time	T_i (°C)	
	Swollen-monodomain (xLCE-BP)	
	Heating (5°C/min)	Coolimg (5°C/min)
0d	114	108
3d	133	122
5d	137	124
10d	153	134
15d	161	140
20d	170	145
25d	172	148
30d	176	151
35d	180	151
40d	176	158
45d	177	155
50d	178	167

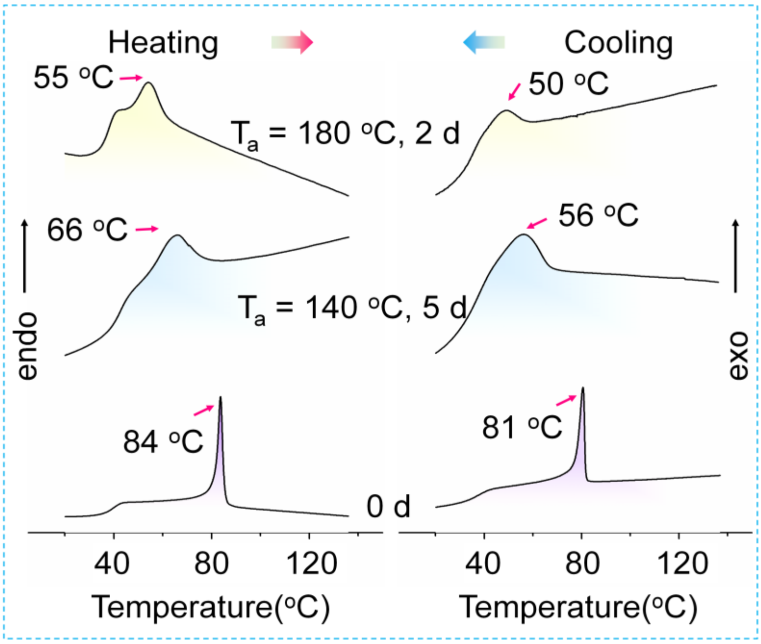

Figure S9. DSC traces of polydomain xLCE-DHMS after annealing at different temperatures.

Table S6 Detailed data obtained from the integrated 1D X-ray diffraction profiles of polydomain xLCE-BP (T_{i0}=114°C) after annealing at 110°C/140°C/180°C for different time.

Annealing time	2θ (deg.)	d (Å)	Intensity (arb.units)	2θ (deg.)	d (Å)	Intensity (arb.units)	2θ (deg.)	d (Å)	Intensity (arb.units)
110°C 0d	2.99	29.50	0.06	5.93	14.88	0.17	20.25	4.38	0.32
110°C 3d	3.05	28.94	0.06	5.97	14.80	0.18	20.25	4.38	0.34
110°C 5d	3.11	28.40	0.07	6.02	14.66	0.19	20.44	4.34	0.34
110°C 10d	3.11	28.40	0.07	6.04	14.61	0.21	20.44	4.34	0.35
110°C 15d	3.14	28.17	0.07	6.08	14.54	0.19	20.25	4.38	0.32
110°C 20d	3.13	28.23	0.07	6.08	14.54	0.20	20.44	4.34	0.33
110°C 25d	3.15	28.05	0.06	6.11	14.44	0.15	20.44	4.34	0.38
110°C 30d	3.20	27.60	0.07	6.06	14.58	0.19	20.25	4.38	0.35
110°C 35d	3.13	28.23	0.07	6.10	14.49	0.23	20.25	4.38	0.29
110°C 40d	3.16	27.94	0.06	6.08	14.54	0.19	20.25	4.38	0.33
110°C 45d	3.09	28.58	0.07	6.10	14.49	0.20	20.25	4.38	0.35
110°C 50d	3.13	28.23	0.07	6.11	14.44	0.19	20.25	4.38	0.34
140°C 5d	3.15	28.05	0.04	6.20	14.26	0.09	20.11	4.41	0.30
180°C 4d	/	/	/	6.45	13.71	0.06	20.03	4.43	0.28

Table S7 Detailed data obtained from the 1D X-ray diffraction profiles of monodomain xLCE-BP annealed at 110°C for different time and 180°C for 1 day respectively. As the monodomain samples were obtained by annealing the stretched polydomain xLCE-BP, there is no monodomain xLCE-BP at 0d. Therefore, the T_i data of polydomain xLCE-BP before annealing were used for 0d.

Annealing time	2θ (deg.)	d (Å)	Intensity (arb.units)	2θ (deg.)	d (Å)	Intensity (arb.units)	2θ (deg.)	d (Å)	Intensity (arb.units)	2θ (deg.)	d (Å)	Intensity (arb.units)	2θ (deg.)	d (Å)	Intensity (arb.units)
110°C 0d	2.99	29.50	0.06	5.93	14.88	0.17							20.25	4.38	0.32
110°C 3d	2.70	29.05	0.06	5.95	14.83	0.31	9.23	9.58	0.02	/	/	/	20.26	4.38	0.22
	3.04	32.86	0.17	6.16	14.33	0.37	/	/	/	/	/	/	/	/	/
110°C 5d	2.68	29.12	0.10	5.95	14.83	3.30	9.12	9.69	0.03	12.08	7.32	0.03	20.29	4.37	0.21
	3.01	30.76	0.27	6.16	14.35	2.69	/	/	/	/	/	/	/	/	/
110°C	2.88	28.03	0.15	6.00	14.72	1.34	9.07	9.75	0.03	11.83	7.48	0.03	20.31	4.37	0.23
10d	3.13	29.76	0.21	/	/	/	9.28	10.75	0.02	/	/	/	/	/	/
110°C	2.94	27.23	0.19	6.04	14.61	1.11	9.16	9.65	0.03	12.11	7.30	0.03	20.27	4.38	0.22
15d	3.24	29.00	0.16	/	/	/	8.85	10.65	0.02	/	/	/	/	/	/
110°C	3.04	/	0.26	6.03	14.65	2.21	9.01	9.81	0.03	12.08	7.32	0.03	20.19	4.39	0.21
20d	/	30.00	/	/	/	/	/	/	/	/	/	/	/	/	/
110°C	3.00	27.35	0.17	6.06	14.58	0.38	9.01	9.81	0.02	/		/	20.17	4.40	0.21
25d	3.24	30.30	0.17	/	/	/	/	/	/	/	/	/	/	/	/
110°C	2.91	27.81	0.26	6.08	14.52	1.27	9.27	9.54	0.03	12.18	7.26	0.03	20.17	4.40	0.22
30d	3.14	28.82	0.35	/	/	/	9.07	10.54	0.03	/	/	/	/	/	/
110°C	3.09	/	0.25	5.96	14.83	0.77	9.13	9.68	0.03	12.22	7.24	0.03	20.28	4.38	0.22
35d	/	30.00	/	6.17	14.31	1.00	/	/	/	/	/	/	/	/	/
110°C	2.94	27.23	0.40	6.12	14.43	1.04	9.11	9.70	0.03	/		/	20.29	4.37	0.21
40d	3.24	30.61	0.37	/	/	/	/	/	/	/	/	/	/	/	/
110°C	2.91	28.20	0.20	6.10	14.49	1.32	9.28	9.53	0.03	12.24	7.23	0.03	20.32	4.37	0.23
45d	3.13	30.45	0.29	/	/	/	9.11	10.53	0.03	/	/	/	/	/	/
110°C	2.91	28.37	0.27	6.00	14.72	0.55	9.28	9.53	0.02	/	/	/	20.17	4.40	0.21
50d	3.13	29.61	0.41	/	/	/	9.06	10.53	0.02	/	/	/	/	/	/
180°C 1d			0.04	6.13	14.40	0.16	/	/	/	/	/		20.02	4.43	0.22

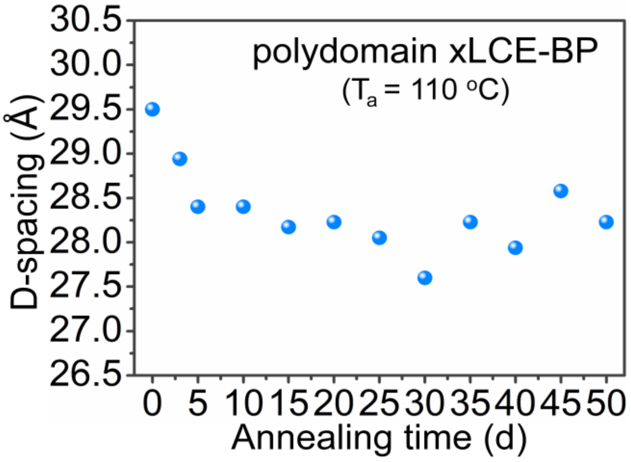

Figure S10. The change of d-spacing of polydomain of xLCE-BP (T_{i0} = 114°C) after annealing at 110°C from 0d to 50d.

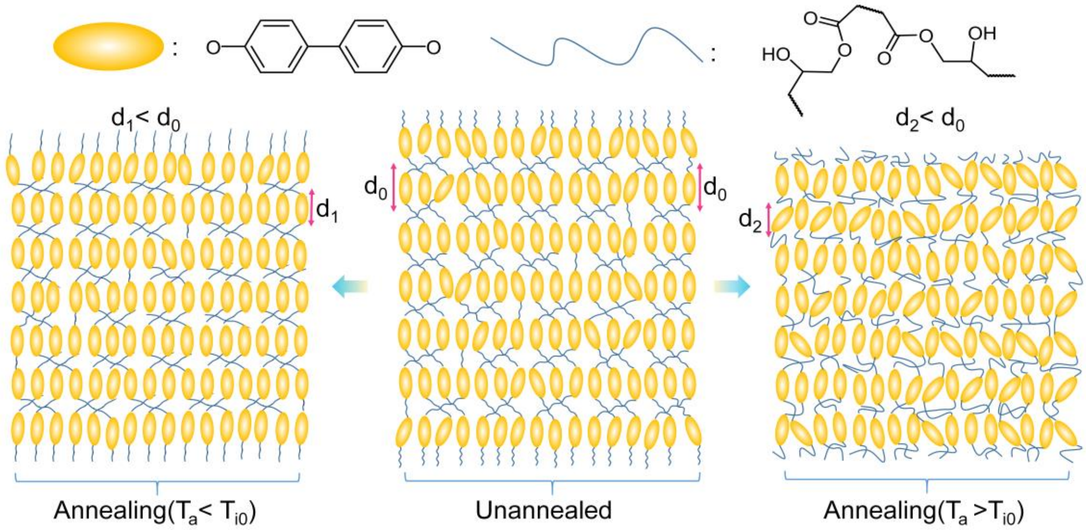

Figure S11. Illustration on the structural change of xLCE-BP after annealing。

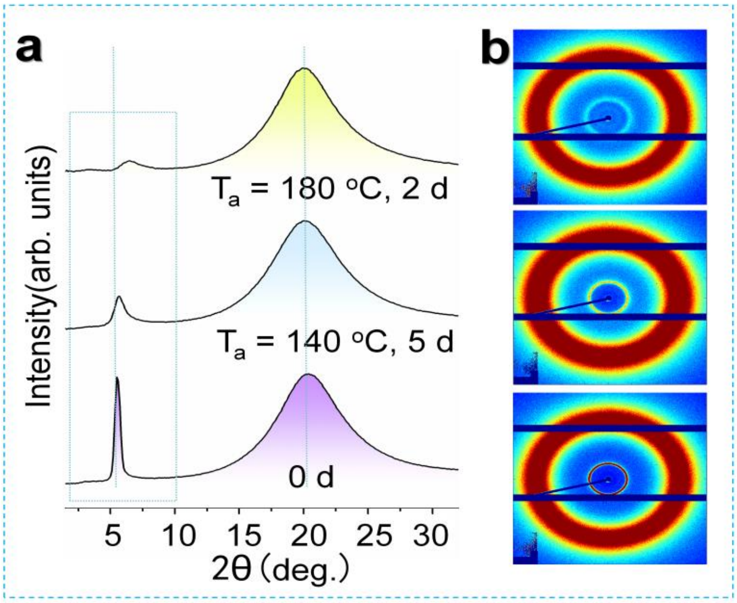

Figure S12. (a) Integrated 1D X-ray diffraction profiles of polydomain xLCE-DHMS after annealing at different temperatures; (b) The X-ray images of the original polydomain xLCE-DHMS and the sample after annealing at 140°C for 5 d and annealing at 180°C for 2 d.

Table S8 Detailed data obtained from the integrated 1D X-ray diffraction profiles of polydomain xLCE-DHMS after annealing at different temperatures for different time.

Annealing Temperature & time	2θ (deg.)	d (Å)	Intensity (arb.units)	2θ (deg.)	d (Å)	Intensity (arb.units)
unanealed	5.56	15.87	0.28	20.41	4.35	0.28
140ºC 5d	5.67	15.59	0.11	20.10	4.14	0.30
180ºC 2d	6.54	13.51	0.06	20.05	4.43	0.27

References

1. Pei Z. Q, Yang Y, Chen Q. M, Terentjev EM, Wei Y, Ji Y. Mouldable liquid-crystalline elastomer actuators with exchangeable covalent bonds. Nat. Mater. **13**, 36-41 (2014).

2. Chen Q. M, Li Y. S, Yang Y, Xu Y. S, Qian X. J, Wei Y, Ji Y. Durable liquid-crystalline vitrimer actuators. Chem Sci **10**, 3025-3030 (2019).

3. Wu Y. H, Zhang S, Yang Y, Li Z, Wei Y, Ji Y. Locally controllable magnetic soft actuators with reprogrammable contraction-derived motions. Sci. Adv. **8**, (2022).

4. Chen Q, Li W, Wei Y, Ji Y. Reprogrammable 3D Liquid-crystalline actuators with precisely controllable stepwise actuation. Adv. Intell. Syst. **3**, 2000249 (2021).

5. Pei Z. Q, Yang Y, Chen Q. M, Wei Y, Ji Y. Regional shape control of strategically assembled multishape memory vitrimers. Adv. Mater. **28**, 156-160 (2016).

6. Zhang S, Zhang Y. B, Wu Y. H, Yang Y, Chen Q. M, Liang H, Wei Y, Ji Y. A magnetic solder for assembling bulk covalent adaptable network blocks. Chem Sci **11**, 7694-7700 (2020).

7. Zhang J, Chen Y, Brook M. A. Facile functionalization of PDMS elastomer surfaces using thiol ene click chemistry. Langmuir **29,** 12432-12442 (2013).
