# Supplementary material for: Enabling liquid crystal elastomers with tunable actuation temperature
Source: Nat Commun. 2023 Jun 14;14:3518. doi: 10.1038/s41467-023-39238-2 (PMC10267162; doi:10.1038/s41467-023-39238-2)
Supplement: Supplementary file 3 — Description of Additional Supplementary Files [file 41467_2023_39238_MOESM3_ESM.pdf]

File Name: Supplementary Movie 1
Description: A heating-cooling cycle of monodomain xLCE-BP.
